# Effect of allergen-specific immunotherapy with purified Alt a1 on AMP responsiveness, exhaled nitric oxide and exhaled breath condensate pH: a randomized double blind study

**DOI:** 10.1186/1710-1492-6-27

**Published:** 2010-09-16

**Authors:** Luis Prieto, Ricardo Palacios, Dulce Aldana, Anna Ferrer, Carmen Perez-Frances, Victoria Lopez, Rocio Rojas

**Affiliations:** 1 Departamento de Medicina, Universidad de Valencia, Valencia, Spain; 2 Diater Laboratorios SA, Madrid, Spain

## Abstract

**Background:**

Little information is available on the effect of allergen-specific immunotherapy on airway responsiveness and markers in exhaled air. The aims of this study were to assess the safety of immunotherapy with purified natural Alt a1 and its effect on airway responsiveness to direct and indirect bronchoconstrictor agents and markers in exhaled air.

**Methods:**

This was a randomized double-blind trial. Subjects with allergic rhinitis with or without mild/moderate asthma sensitized to *A alternata *and who also had a positive skin prick test to Alt a1 were randomized to treatment with placebo (n = 18) or purified natural Alt a1 (n = 22) subcutaneously for 12 months. Bronchial responsiveness to adenosine 5'-monophosphate (AMP) and methacholine, exhaled nitric oxide (ENO), exhaled breath condensate (EBC) pH, and serum Alt a1-specific IgG_4 _antibodies were measured at baseline and after 6 and 12 months of treatment. Local and systemic adverse events were also registered.

**Results:**

The mean (95% CI) allergen-specific IgG_4 _value for the active treatment group increased from 0.07 μg/mL (0.03-0.11) at baseline to 1.21 μg/mL (0.69-1.73, P < 0.001) at 6 months and to 1.62 μg/mL (1.02-2.22, P < 0.001) at 12 months of treatment. In the placebo group, IgG_4 _value increased nonsignificantly from 0.09 μg/mL (0.06-0.12) at baseline to 0.13 μg/mL (0.07-0.18) at 6 months and to 0.11 μg/mL (0.07-0.15) at 12 months of treatment. Changes in the active treatment group were significantly higher than in the placebo group both at 6 months (P < 0.001) and at 12 months of treatment (P < 0.0001). However, changes in AMP and methacholine responsiveness, ENO and EBC pH levels were not significantly different between treatment groups. The overall incidence of adverse events was comparable between the treatment groups.

**Conclusion:**

Although allergen-specific immunotherapy with purified natural Alt a1 is well tolerated and induces an allergen-specific IgG_4 _response, treatment is not associated with changes in AMP or methacholine responsiveness or with significant improvements in markers of inflammation in exhaled air. These findings suggest dissociation between the immunotherapy-induced increase in IgG_4 _levels and its effect on airway responsiveness and inflammation.

## Background

Airway inflammation plays a central role in the pathogenesis of asthma and is associated with an increase in airway responsiveness to various spasmogens[1]. Clinically and for research purposes, airway responsiveness is measured by bronchial challenge, usually with methacholine or histamine[2]; however, adenosine 5'-monophosphate (AMP) has been introduced as a bronchoconstrictive stimulus more recently. Whereas histamine and methacholine act by a direct effect on the relevant receptors on airway smooth muscle stimulating airway muscle contraction directly, AMP-induced bronchoconstriction occurs predominantly indirectly, causing "primed" mast cells degranulation and the release of histamine and other mediators with subsequent smooth muscle contraction[3,4]. It has been suggested that the bronchial responsiveness to inhaled AMP may reflect changes in airway inflammation induced by allergen exposure[5,6] or by allergen immunotherapy[7] with greater precision and sensitivity than the response to direct bronchoconstrictor agents.

Increased concentrations of exhaled nitric oxide (ENO)[8,9] and acidification of exhaled breath condensate (EBC)[10,11] have been demonstrated in asthma. In addition, both ENO and EBC pH are correlated with the number of eosinophils in the lower respiratory tract [11,12]. Therefore, these parameters have been proposed as markers of airway inflammation and disease severity in asthma[13,14].

*Alternaria alternata *is considered one of the most important aeroallergens in the United States[15,16] and in Europe[17]. Moreover, sensitization to *A alternata *has been associated with severe cases of asthma and respiratory arrest[17]. One of the major difficulties for allergen-specific immunotherapy (SIT) with fungal extracts arises from the variability and complexity of fungal organism, with the subsequent difference in composition and allergenic potency of commercial extracts[18,19]. Although *A alternata *contains several different allergens, Alt a1 represents by far the most important with greater than 90% of sensitized individuals having IgE antibody against this allergen[20]. Therefore, immunotherapy with Alt a1 alone may well suffice to improve manifestations of sensitization to the entire allergen composition of *A alternata*. The mechanism of action of SIT is not definitively established, but it might be consequence of treatment-induced changes on the underlying immunological mechanisms with the subsequent beneficial effect on allergen-induced airway inflammation[21,22]. Thus, the identification of the effect of SIT on airway responsiveness and inflammation might represent a relevant support to the efficacy of treatment in clinical studies. The effect of SIT on airway responsiveness to direct bronchoconstrictor agents has been determined in a limited number of controlled studies, and the results of these investigations have been inconsistent[23-31]. To the best of our knowledge, however, only two studies have determined the effect of SIT on airway responsiveness to indirect bronchoconstrictor agents such as cold dry air[32] and inhaled AMP[7]. Additionally, little is known about the effect of SIT on ENO[33,34] and no information is available about the effect of SIT on EBC pH.

The aims of this pilot study were to determine the safety of SIT with purified natural Alt a1 and to evaluate its effects on airway responsiveness and inflammatory markers in exhaled air and EBC in subjects with respiratory allergy (allergic rhinitis with or without asthma) sensitized to this allergen. The primary outcomes were the airway responsiveness to AMP, ENO values and side effects. Secondary outcomes included lung function, airway responsiveness to methacholine, and EBC pH.

## Methods

### Subjects

Male and nonpregnant female subjects 9 - 60 yrs of age with allergic rhinitis, with or without mild/moderate asthma, and skin sensitization to both *A alternata *and Alt a1 (3 μg/mL, Diater Laboratories, Madrid, Spain) were recruited from the allergy clinic of our institution. Sensitization was confirmed by skin prick test (weal ≥3 mm) with both *A alternata *extract and purified natural Alt a1 (Diater Laboratories S A, Madrid, Spain). Asthma was identified by the presence of symptoms of wheeze, breathlessness and cough plus methacholine airway hyperresponsiveness with a PC_20 _(provocative concentration required to produce a 20% fall in FEV_1_) of less than 8 mg/ml if the FEV_1_/FVC was 70% or greater or an improvement of the FEV_1 _from predicted of 15% or greater after 200 μg of inhaled salbutamol if the FEV_1_/FVC was less than 70%. Subjects with allergic rhinitis were defined as those individuals with a characteristic history of rhinitis (rhinorrhea, sneezing, obstruction, and pruritus). All asthmatic subjects were well-controlled for at least 3 months by treatment with inhaled β_2 _agonists on demand or with a daily dose of beclomethasone dipropionate ≤1000 μg or equivalent. In the 3 months before the study, patients had asthma symptoms no more than twice a week, did not wake at night because of asthma and did not suffer asthma exacerbation. They had no changes in their dose of inhaled corticosteroids (ICS) in the last 3 months, and FEV_1 _al baseline had to be >70% of predicted. All patients were nonsmokers, and none had history of chronic bronchitis, emphysema, or respiratory tract infections during the 4 weeks before the study. Current smokers and patients with significant renal, hepatic, or cardiovascular disease were specifically excluded. The study protocol (DIA-ALE-2004-01) was approved by the ethics committee of the Hospital Universitario Dr Peset and the health authorities. Written informed consent was obtained from each patient or their parents before participation.

### Study design

This was a single-center, randomized, double-blind, placebo-controlled, parallel-group study. Upon entry of patients into the study, a detailed history was taken and physical examination, spirometry, ENO, and bronchial challenges with methacholine and AMP were carried out; EBC and blood samples were also obtained. Methacholine and AMP challenges were conducted on separate days with the order of challenge randomized. Patients were then randomized to receive either active treatment consisting of increasing doses of purified natural Alt a1 adsorbed in aluminium hydroxide (Diater SA, Madrid, Spain) given subcutaneously, followed by monthly maintenance treatment or placebo (aluminium hydroxide gel). A maintenance dose of 0.2 μg was achieved in all participants. Extracts for immunotherapy were reconstituted on the day of administration and single-dose vials were used. Patients returned to the laboratory after 6 and 12 months of treatment. In each period, the same determinations performed at baseline were repeated.

The dose of intranasal or ICS (if used) was maintained unchanged during the study. Salbutamol metered-dose inhaler, oral antihistamines and intranasal antihistamines were used on an "as-needed" basis to control pulmonary or nasal symptoms, respectively. No other medications were allowed to be used during the study. Subjects were asked not to take ICS for 12 hours, salbutamol for at least 6 hours, oral antihistamines for at least 72 hours and intranasal antihistamines for at least 24 hours before each study visit.

### Study outcome variables

#### Inhalation challenge tests

Lung function was measured using a calibrated pneumotachograph (Jaeger MasterScope; Erich Jaeger GmbH; Würzburg, Germany) according to standardized guidelines[35]. Inhalation provocation tests were performed using a modification of the dosimeter method[36] as previously reported[37,38]. Methacholine (Provocholine, Diater SA, Madrid Spain) and AMP (Sigma Chemical; St Louis, MO, USA) were dissolved freshly in 0.9% saline solution to produce doubling concentration ranges of 0.095 to 25 mg/ml for methacholine and from 0.39 to 400 mg/ml for AMP. Subjects inhaled the aerosolized methacholine and AMP solutions (Mefar; Brescia, Italy) in five respiratory capacity inhalations. The nebulizer output was 10 μl per breath. The test was interrupted when a 20% decrease in FEV_1 _from the post-saline solution administration value was recorded or when the highest concentration was administered.

#### ENO measurement technique

Measurements were performed before spirometry and challenge tests in accordance with the American Thoracic Society/European Respiratory Society recommendations[39], with a portable device (NIOX-MINO, Aerocrine AB, Stockholm, Sweden) and defined in parts per billion (ppb).

#### Collection of EBC

EBC was collected using the RTube collection system (Respiratory Research, Inc, Charlottesville, VA) as previously reported[40]. Aluminium sleeves for RTubes were kept for at least 1 h in a freezer consistently at -20°C before use. Subjects breathed normally through their mouth into the device for 15 min and they were also instructed to temporarily discontinue collection if they needed to swallow saliva or cough. Nose clips were not worn. At the end of collection, the sample was carefully removed from the collection system and EBC pH was determined in a 0.2 ml aliquot immediately after collection.

#### Measurement of EBC pH

The pH of the EBC was measured after deaeration with argon using a calibrated pH meter incorporating a sensor with temperature compensation (model pH 900) with a Biotrode electrode (Metrohm AG, Herisau, Switzerland), and with an accuracy of ± 0.01 pH. Deaeration was performed by bubbling argon through the sample for 8 min[40,41].

#### Measurement of serum rAlt a1-specific IgG_4_

Specific IgG_4 _levels to rAlt a1 were evaluated by means of the Fluoro enzyme immunoassay (FEIA), following the instructions of ImmunoCap Specific IgG and IgG_4 _(Phadia AB, Uppsala, Sweden).

#### Adverse events

Details of adverse events were collected during the study on a form that recorded all events, irrespective of suspected relationship to the study medication and of mild, moderate or serious severity.

### Specific immunotherapy

*Alternaria alternata *extract and nAlt a1 were produced by Diater (Madrid, Spain). Raw material containing spores and mycelia of *Alternaria Alternata *(CBS 103.33) was purchased from Allergon (Engelholm, Sweden). Extraction was performed in PBS buffer for 2 hours at 4°C. After centrifugation (4500 g, 30 min) the supernatant was filtered, subjected to diafiltration (cut-off 5000 Da) and lyophilized. Alt a1 was purified from *A alternata *extract by three chromatographic steps. Briefly, *A alternata *extract was reconstituted in starting buffer (Bis-Tris pH 6.5) and the solution was separated by anion exchange chromatography (Hitrap Q XL; GE Healthcare, Uppsala, Sweden). The first peak was collected, desalted and applied to Hitrap SP FF cation exchange column (GE Healthcare, Uppsala, Sweden) equilibrated with acetate buffer pH 5.2. The flow-through fraction was desalted and separated by gel filtration chromatography (Superdex 75 prep grade; GE Healthcare, Uppsala, Sweden) using ammonium bicarbonate buffer. The fraction containing Alt a1 was lyophilized. A full characterization to Alt a1 was performed before manufacturing the vaccines (data not shown).

SIT was administered with a cluster schedule that made it possible to reach the maintenance dose in 4 weeks. Both placebo and Alt a1 extract were administered in an identical fashion. Maintenance injections were administered every 4 weeks for 1 year (Table 1).

**Table 1:** Cluster schedule administered during SIT.

Day	Interval	Vial	Dose (mL)	Allergen dose (mcg/mL)
1		2	0.1 + 0.2	0.0025 + 0.005
8	Weekly	2	0.4 + 0.4	0.01 + 0.01
15	Weekly	3	0.1 + 0.2	0.025 + 0.05
22	Weekly	3	0.4 + 0.4	0.1 + 0.1
37	Fortnightly	3	0.8	0.2
67	Monthly	3	0.8	0.2
97	Monthly	3	0.8	0.2
...*	Monthly	3	0.8	0.2

*Monthly doses were administered until the last dose (Vial 3, 0.8 mL)

### Skin prick tests

Skin-prick testing was performed with glycerinated saline (negative control), histamine (1% histamine dihydrochloride, positive control), and house dust mites (Dermatophagoides pteronyssinus and D. farinae), household pets (cat and dog), pollens (mixed grass, olive, Parietaria judaica, Artemisia, Platanus orientalis, Cupresus arizonica and Salsola kali), and moulds (*Alternaria alternata*, Aspergillus fumigatus, Cladosporium and Penicillium). Furthermore, skin-prick testing was performed with a standardized extract of purified natural Alt a1 (3 μg/ml). After 20 min, weal size was recorded as the long axis and its perpendicular. A skin-test response was regarded as positive if the weal was ≥3 mm larger in diameter than that of the glycerinated saline.

### Data analysis

An intention-to-treat approach was followed in the analysis of efficacy data. All patients with a baseline and at least one postrandomization measurement were included in the efficacy analysis according to the group to which they were randomized. The safety population comprised all patients who received at least one dose of SIT or placebo.

To calculate a continuous index of methacholine and AMP responsiveness, the bronchial responsiveness index (BRI) was calculated, using the method described by Burrows et al[42] as the percentage decline in FEV_1 _divided by the log of the last concentration of agonist, expressed in mg/dL. All ENO values were log-transformed before analysis and are presented as geometric means with 95% confidence intervals (CI). All other numerical variables are reported as arithmetic means with 95% CI.

The primary outcomes of the study were the BRI to AMP and ENO concentration. On the basis of previous data[43], this study had 80% power to detect a difference of 1.5%/log mg/dL in the BRI to AMP and >90% power to detect a difference of 7 ppb in the ENO values between the two groups.

Data were analyzed using a standard statistical software package (InStat for Windows version 3.0; GraphPad Software Inc, San Diego, CA, USA). Comparisons of the baseline characteristics of the two groups were performed by unpaired Student's t test for continuous data and by Fisher's exact tests for categorical data. Comparisons of treatment effects of placebo and SIT on BRI to methacholine, BRI to AMP, FEV_1_, ENO, EBC pH and serum Alt a1 specific IgG_4 _were made using two-factor repeated-measures analysis of variance to analyze the effect of the two independent variables, treatment and time, on the outcome variables described previously. Correlations between variables were calculated with Pearson correlation coefficient. All comparisons were two-tailed, and P values less than 0.05 were considered significant.

## Results

Forty-two subjects were enrolled, and 40 were assigned randomized treatment sets and included in the safety evaluation. One subject (SIT group) discontinued prematurely before the first visit after randomization due to an adverse event (local pain in the injection area without inflammatory signs after the two first doses of SIT), thus leaving 21 active treatment and 18 placebo subjects for analysis at 6 months. Fourth patients (SIT group) declined the previous acceptance for participation after 6 months of treatment for social problems not related to the treatment. Thus, 35 patients (17 in the actively treated group and 18 in the placebo group) completed 12 months of treatment. Baseline characteristics were comparable for the two treatment groups (table 2).

**Table 2:** Baseline characteristics of the two treatment groups.

	SIT group (n = 21)	Placebo group (n = 18)	P
Age*, years	25 (22-29)	22 (18-26)	0.21
Sex (male/femle)	9/12	13/5	0.11
Asthma + rhinitis/rhinitis only	13/8	10/8	0.75
Sensitization to other allergens (yes/no)	17/4	15/3	0.95
ICS treatment (yes/no)	11/10	9/9	0.98
ICS dose* (beclomethasone equivalent), μg/day	392 (268-535)	456 (310-620)	0.51
FEV_1_*, % predicted	95.4 (89.8-101.0)	99.3 (92.4-106.2)	0.36
FEV_1_/FVC*, %	78.8 (74.9-82.6)	80.9 (76.6-85.2)	0.44
BRI*, %/log mg/dl			
Methacholine	7.2 (4.3-10.1)	7.1 (4.7-9.5)	0.95
AMP	3.6 (2.3-5.0)	3.7 (2.1-5.3)	0.93
ENO**, ppb	38.5 (27.3-54.6)	40.4 827.2-60.1)	0.85
EBC pH*	7.43 (7.15-7.70)	7.50 (7.24-7.77)	0.68

Beclomethasone dipropionate equivalent dose of ICS was calculated on the basis of fluticasone propionate being twice as potent as beclomethasone dipropionate or budesonide, so that the equivalent fluticasone propionate dose was multiplied 2-fold; *Data are given as means (95% confidence interval); **Data are given as geometric mean (95% confidence interval). Abbreviations: AMP = adenosine 5'monophosphate; BRI = bronchial responsiveness index; ENO = exhaled nitric oxide; FEV_1_= forced expiratory volume in 1 second; FVC = forced vital capacity; EBC = exhaled breath condensate.

### AMP responsiveness

**Figure 1:**
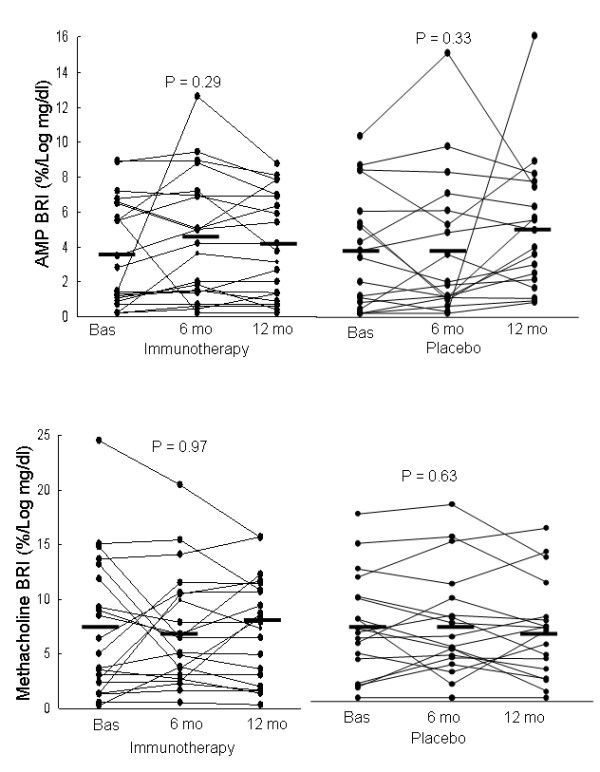
**Individual values for the adenosine 5'-monophosphate (AMP) and methacholine bronchial responsiveness index (BRI) in the specific immunotherapy and placebo groups at baseline and after 6 and 12 months of treatment**. Horizontal lines are geometric means. Changes in AMP BRI values were not significantly different between the SIT and placebo groups (P = 0.35 and P = 0.50 for changes at 6 and 12 months of treatment, respectively). Furthermore, changes in methacholine BRI values were not significantly different between the SIT and placebo groups (P = 0.92 and P = 0.61 for changes at 6 and 12 months of treatment, respectively).

In both groups, changes from baseline in BRI values after 6 and 12 months of treatment were not significant (Table 3 and Figure 1). Furthermore, changes in AMP BRI values were not significantly different between the SIT and placebo groups, the mean difference being -0.8%/log mg/dL (-2.5 to 0.9, P = 0.35) and 0.7%/log mg/dL (-1.3 to 2.6, P = 0.50) after 6 and 12 months of treatment, respectively.

**Table 3:** Changes in FEV_1 _and in methacholine and AMP responsiveness in the SIT and placebo groups.

	Baseline	6 months	12 months
SIT group			
FEV_1_, L	3.31 (2.99-3.63)	3.32 (3.01-3.64)	3.31
Methacholine BRI, %/log mg/dl	7.2 (4.3-10.1)	7.2 (4.8-9.5)	7.4 (5.2-9.5)
AMP BRI, %/log mg/dl	3.6 (2.3-5.0)	4.5 (2.9-6.1)	4.1 (2.7-5.4)
Placebo group			
FEV_1_, L	3.69 (3.21-4.17)	3.59 (3.15-4.03)	3.64 (3.20-4.07)
Methacholine BRI, %/log mg/dl	7.1 (4.7-9.7)	7.2 (4.7-9.7)	6.6 (4.4-8.9)
AMP BRI, %/log mg/dl	3.7 (2.1-5.3)	3.8 (1.8-5.7)	4.8 (3.0-6.6)

Values are expressed as mean (95% confidence interval). For abbreviations see Table 2.

### Exhaled nitric oxide

**Figure 2:**
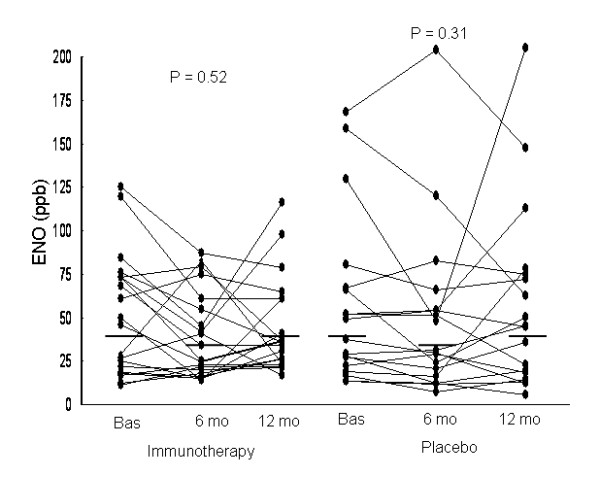
**Individual values for exhaled nitric oxide (ENO) concentrations in the specific immunotherapy and placebo groups at baseline and after 6 and 12 months of treatment**. Horizontal lines are geometric means. Changes in ENO levels were not significantly different between the SIT and placebo groups (P = 0.86 and P = 0.53 for changes at 6 and 12 months of treatment, respectively).

In both groups, changes from baseline in ENO values after 6 and 12 months of treatment were not significant (Table 4 and Figure 2). Furthermore, changes in ENO were not significantly different between the SIT and placebo groups, the mean difference being -1.5 ppb (-18.5 to 15.6, P = 0.86) and -6.4 ppb (-26.9 to 14.0, P = 0.53) after 6 and 12 months of treatment, respectively.

**Table 4:** Changes in exhaled nitric oxide (ENO), exhaled breath condensate (EBC) pH, and Alt a1-specific IgG_4 _in serum.

	Baseline	6 months	12 months
SIT group			
ENO, ppb	38.5 (27.3-54.6)	33.4 (25.2-44.4)	37.7 (29.5-48.0)
P		NS	NS
EBC pH	7.43 (7.15-7.70)	6.91 (6.55-7.28)	7.57 (7.34-7.79)
P		< 0.05	NS
Alt a1-specific IgG_4_, μg/ml	0.07 (0.03-0.11)	1.21 (0.69-1.73)	1.62 (1.02-2.22)
P		< 0.001	< 0.001
Placebo group			
ENO, ppb	40.4 (27.2-60.1)	32.1 (20.7-49.8)	37.0 (22.9-59.2)
P		NS	NS
EBC pH	7.50 (7.24-7.77)	7.28 (6.97-7.59)	7.44 (7.21-7.67)
P		NS	NS
Alt a1-specific IgG_4_, μg/ml	0.09 (0.06-0.12)	0.13 (0.07-0.18)	0.11 (0.07-0.15)
P		NS	NS

Values are geometric means (95% confidence interval) for ENO and means (95% confidence interval) for EBC pH and Alt a1-spècific IgG_4 _values. P values are for the comparison with baseline values within groups. For abbreviations see Table 2.

### Lung function

In both groups, changes from baseline in FEV_1 _values were not significant (Table 3). Furthermore, changes in FEV_1 _values were not significantly different between the SIT and placebo groups, the mean difference being 0.12 L (95% CI, -0.06 to 0.29, P = 0.18) at 6 months and 0.06 L (-0.09 to 0.21, P = 0.91) at 12 months of treatment.

### Methacholine responsiveness

In both groups, changes in these values were not significant (Table 3 and Figure 1). Furthermore, changes in methacholine BRI values were not significantly different between the SIT and placebo groups, the mean difference being 0.1%/log mg/dL (-2.4 to 2.7, P = 0.92) and -0.7%/log mg/dL (-3.2 to 1.9, P = 0.61) after 6 and 12 months of treatment, respectively.

### Exhaled breath condensate pH

**Figure 3:**
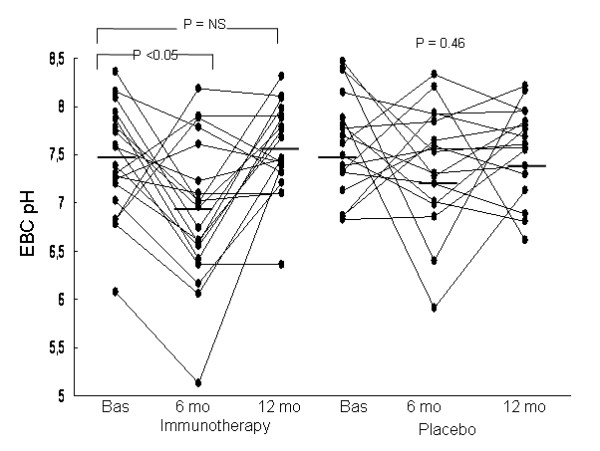
**Individual values for exhaled breath condensate (EBC) pH in the specific immunotherapy and placebo groups at baseline and after 6 and 12 months of treatment**. Horizontal lines are means. Changes in EBC pH were not significantly different between the SIT and placebo groups (P = 0.30 and P = 0.33 for changes at 6 and 12 months of treatment, respectively).

The pH of EBC was not performed in 3 subjects (2 in the SIT group and 1 in the placebo group) due to technical problems. Thus EBC pH could be compared in 36 subjects (19 in the SIT group and 17 in the placebo group). In the SIT group, EBC pH values decreased significantly (Table 4 and Figure 3) in the evaluation performed after 6 months of treatment (P < 0.05), but not in the final evaluation performed after 12 months of treatment. These changes did not reach significance at any time point in the placebo group. Furthermore, changes in EBC pH were not significantly different between the SIT and placebo groups, the mean difference being 0.30 (-0.28 to 0.88, P = 0.30) and -0.20 (-0.62 to 0.21, P = 0.33) after 6 and 12 months of treatment, respectively.

### Alt a1-specific IgG_4_

Active treatment induced strong IgG_4 _responses against the Alt a1 allergen (Table 4). IgG_4 _concentrations increased approximately 17-fold after 6 months of treatment and 23-fold after 12 months of treatment. Comparison between the groups showed statistically significant differences at all time points after the commencement of treatment, the mean difference being 1.10 μg/mL (95% CI, 0.63 to 1.57, P < 0.001) and 1.53 μg/mL (95% CI, 0.96 to 2.09, P < 0.0001) after 6 and 12 months of treatment, respectively.

### Safety and tolerability

SIT with Alt a1 was well tolerated, with no life-threatening reactions. The overall incidence of adverse events was comparable between the treatment groups. There were 33 local adverse events, 17 and 16 in the active and placebo groups, respectively. These episodes of pruritis, pain or swelling were expected consequent to subcutaneous allergen or histamine injection. They were well tolerated with symptomatic treatment with antihistamines and ice. Thirty-one adverse events were classified as systemic, with 15 and 16 in the active and placebo groups, respectively. Most were episodes of rhinoconjunctivitis (3 in the active group and 2 in the placebo group), asthma exacerbation (4 in the active group and 3 in the placebo group) or common cold (4 in the active group and 6 in the placebo group). There were 5 episodes of general urticaria or pruritis (2 in the active group and 3 in the placebo group). All these systemic adverse events were considered by the investigator as not related with the study treatment.

### Correlations

At baseline, a significant correlation was found between methacholine and AMP BRI values (r = 0.80, P < 0.0001) as well as between BRI to AMP and ENO (r = 0.38, P = 0.02). There was also a significant correlation between SIT induced changes in methacholine and AMP responsiveness (r = 0.68, P = 0.0007 and r = 0.43, P = 0.05 for changes at 6 and 12 months of treatment, respectively). No other correlations were detected.

## Discussion

This first clinical study of immunotherapy using a purified natural Alt a1 (the major *A alternata *allergen) for the treatment of allergic rhinitis with or without asthma demonstrated the good tolerance of the preparation, together with the induction of strong allergen-specific IgG4 antibody responses. However, the treatment was not associated with significant reductions in methacholine and AMP responsiveness or with significant modifications of ENO and EBC pH values. These findings suggest that SIT-induced changes in allergen-specific IgG_4 _concentrations are not necessarily associated with improvements in airway responsiveness, ENO or EBC pH values.

At the time of writing, there has been only one published study[7] on the effect of SIT on airway responsiveness to AMP. In a group of non-asthmatic subjects with allergic rhinitis monosensitized to *Parietaria judaica*, Polosa et al[7] reported that SIT with Parietaria pollen extract for two years was associated with a significant protection against the seasonal deterioration of airway responsiveness to AMP, whereas no significant effect was observed on bronchial hyperresponsiveness to methacholine. By contrast, our results demonstrated that AMP responsiveness changed in response to treatment with SIT to a similar extent than did methacholine responsiveness. Differences in patient characteristics, study design, allergen administrated for SIT, duration of treatment and challenge methods between the study by Polosa et al[7] and the present study preclude a proper comparison. However, from our results it is evident that SIT with Alt a1 does not induce significant changes in AMP responsiveness.

On the other hand, SIT appears to have no effect on airway responsiveness to methacholine. This confirms the results of other controlled trials investigating the effect of SIT administered by subcutaneous injection on methacholine responsiveness in subjects with respiratory allergy[7,30,31]. By contrast, other investigations identified a significant improvement in methacholine responsiveness after SIT with house dust mites[23,27] or pollen allergens[24,25]. Reasons to such discrepancies are not evident, but might be related to differences in patients' characteristics, to diversity in the disease activity in the subjects studied, to differences in the characteristics of the allergenic extract administered, or to important differences in the statistical analysis.

It has been hypothesized that SIT results in a deviation in the T lymphocyte response and a modified T_H2 _response. An increase in T-regulatory cells contributes to this process, and their production of IL-10 and TGF-β favors the suppression of IgE production and the increase in IgG_4 _antibodies[44,45]. Additionally, it has been suggested that allergen-specific IgG_4 _antibodies have the potential to reduce early responses to allergen by blocking Fcε-dependent mast cell activation and release of performed mediators[46]. The results of our study clearly demonstrate that SIT with purified natural Alt a1 is associated with a highly significant increase in allergen-specific IgG_4 _level. However, the increase in serum concentrations of allergen-specific IgG_4 _antibodies in our patients was not associated with a decrease in the response to AMP, an indirect bronchoconstrictor that induces obstruction by stimulation of A_2_-purinoceptors on mast cells[3,4]. Furthermore, no significant correlation was detected between SIT-induced modifications in allergen-specific IgG_4 _antibodies and AMP responsiveness. These results suggest that allergen-specific IgG_4 _has no potential to downregulate non IgE-dependent mast cell responses.

In our subjects with respiratory allergy, we did not detect an affect of SIT on ENO levels. These data are consistent with those of two previous studies performed in children[33,34]. However, this is the first study (to our knowledge) to examine the question of an effect of SIT on EBC pH. In the group of subjects treated with SIT, there was a significant decrease in EBC pH, compared with values at baseline, after 6 months of treatment. However, a decrease in EBC pH was also detected in the placebo group, although it did not reach statistical significance. Furthermore, differences in modifications of EBC pH between the two groups were no significant. Therefore, we believe that the decrease in EBC pH might be consequence of unidentified technical or nontechnical factors and that SIT with Alt a1 does not affect EBC pH values, either positively or negatively. Because ENO and EBC pH have been proposed as procedures for the evaluation of airway inflammation[13,14], our results might be interpreted as an additional argument for the absence of effect of SIT on airway inflammation. However, the correlation between ENO and direct measures of airway inflammation have been of relatively small magnitude[12], and therefore the precise mechanism(s) that link(s) nitric oxide with eosinophilic airway inflammation, and whether elevated ENO concentrations are caused by enhanced activity of eosinophils or by enhanced diffusion through the airway wall due to structural damage, remain to be elucidated. In addition, it must be acknowledged that the interpretation of EBC pH is controversial due to technical factors[40,47], and there is a debate as to whether orally collected EBC pH assays reflect acidification of the lower airways[48].

There were some methodological problems and limitations to this study, which are important to consider. First, a significant proportion of our patients were taking inhaled corticosteroids. Given the beneficial effect of inhaled corticosteroids on pulmonary function, airway responsiveness, ENO and EBC pH, it could be argued that the effects of SIT with Alt a1 might be different in subjects not treated concomitantly with ICS. Therefore, it would be of interest to repeat this type of study in steroid-naïve subjects. Second, natural allergen exposure during each study period was not controlled and we cannot discard that the lack of effect of SIT with Alt a1 on airway responsiveness or airway inflammation might be consequence of a low level of natural allergen exposure. Therefore, the resuls of this study might be different in subjects sensitized to *A alternata *tested during a period of high ambiental allergen exposure. Third, our patients had mild airway responsiveness. Therefore, the lack of effect of SIT on methacholine and AMP responsiveness might be consequence of the low degree of airway responsiveness in our population. Finally, it is worth noting that most of our patients with *A alternata *allergy were also sensitized to other perennial or seasonal allergens. This situation closely emulates what might happen in the normal clinical setting in which monosensitization to *A alternata *is exceptionally detected. However, we acknowledge that the results of this study might not be applicable to subjects monosensitized to *A alternata*.

A favourable safety profile was demonstrated. The majority of the reactions involved erythema and swelling in the vicinity of the injection sites consistent with local allergic reactions or mild trauma caused by the aluminum hydroxide suspension. Systemic reactions were infrequent and mild and occurred with similar prevalence in the two groups. Additionally, the fact that all subjects continued therapy with either the same or higher doses without further problems indicates that the preparation is generally well tolerated. By contrast, although a recent study stated that, in subjects monosensitized to *A alternata*, SIT with a standardized extract was well tolerated[19], it has been reported that SIT with a standardized whole extract of *Alternaria *induces systemic reactions in 19% to 40% of patients[49,50] and in 2% of injections[50]. Therefore, it appears that the safety profile of SIT with Alt a1 is superior to that detected with a conventional standardized extract of *Alternaria*.

## Conclusions

Although SIT with Alt a1 is well tolerated and induces an allergen-specific IgG_4 _response, treatment-induced changes in airway responsiveness to direct and indirect bronchoconstrictor agents, ENO and EBC pH values are no significant. These findings should no necessarily be interpreted as demonstrative of the lack of clinical efficacy, because immunotherapy-induced changes in airway responsiveness or in inflammatory markers have correlated poorly with clinical responses to treatment[31,33].

## Competing interests

L Prieto has served as consultant to GSK, Novartis and Stallergenes, and has received grant founding from GSK and Novartis; R Palacios and D Aldana are employees of Diater Laboratorios SA; A Ferrer, C Perez-Frances and R Rojas have no conflict of interest to disclose.

## Authors' contributions

LP and RP devised the idea of the study and designed the methods; DA performed laboratory methods; LP wrote manuscript drafts, was responsible for data management and statistical analyses, and is the guarantor; AF, CPF, VL, RR and LP were responsible for implementing the study. All authors read and approved the final manuscript.
